# Supplementary material for: From net-zero to zero-fossil in transforming the EU energy system
Source: Nat Commun. 2025 Dec 10;16:10700. doi: 10.1038/s41467-025-66682-z (PMC12695931; doi:10.1038/s41467-025-66682-z)
Supplement: Supplementary file 1 — Supplementary Information [file 41467_2025_66682_MOESM1_ESM.pdf]

From net-zero to zero-fossil in transforming the EU energy system

(Supplementary Information)

Felix Schreyer[1,2], Falko Ueckerdt[1], Robert Pietzcker[1], Adrian Odenweller[1,2], Anne Merfort[1,2], Renato Rodrigues[1], Jessica Strefler[1], Fabrice Lécuyer[1] and Gunnar Luderer[1,2]

[1] Potsdam Institute for Climate Impact Research, Member of the Leibniz Association, Potsdam, Germany

[2] Global Energy Systems Analysis, Faculty of Process Science, Technische Universität Berlin, Berlin, Germany

Contents

Supplementary Figures 3
- Evolution and Composition of Final Energy by Sector 3
- Evolution and Composition of Electricity Generation and Demand 4
- Evolution and Composition of Fossil Fuel Demand 5
- Evolution and Composition of Hydrogen Supply and Demand 6
- Demand for Hydrocarbon Fuels in Final Energy by 2050 7
- Evolution and Composition of Total Greenhouse Gas Emissions 8
- Evolution of Total Carbon Dioxide Removal 9
- Composition of Biomass Supply and Demand by 2050 10
- Carbon Capture, Storage and Utilization 11
- Secondary Energy Prices of Fossil Liquid Fuels and E-fuels 13
- Cost Assessments Across Further Sensitivity Scenarios 14
- Final Energy Prices of Relevant Energy Carriers 15

Supplementary Tables 16
- Overview about EU Net-zero Scenarios Investigated by this Study 16
- Scenario Assumptions in Standard and Sensitivity Scenarios 17

Supplementary References 18

Supplementary Figures

Evolution and Composition of Final Energy by Sector

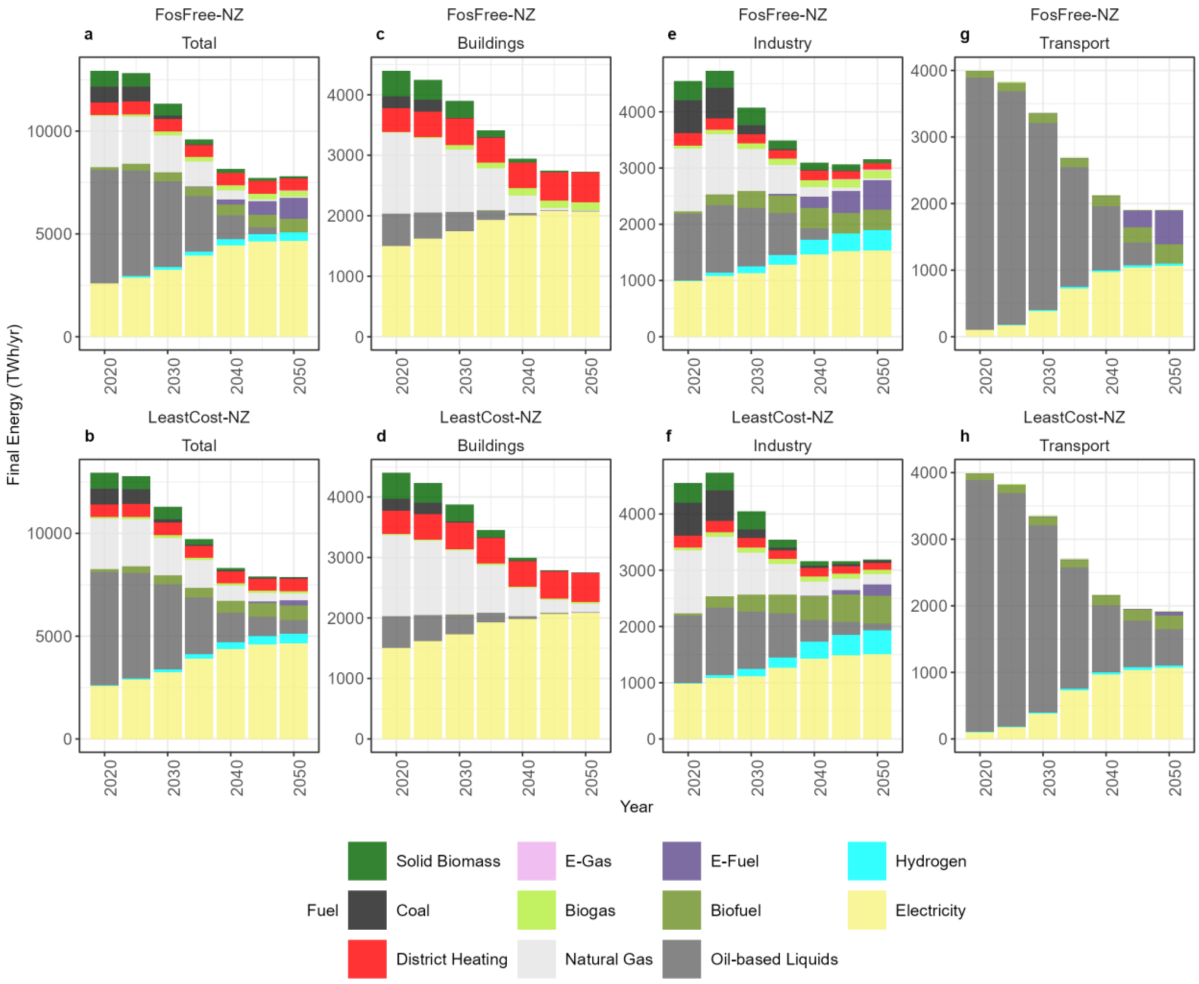

Supplementary Figure 1: EU total final energy demand (panels **a-b**) by energy carrier and sectoral final energy demand for buildings (panels **c-d**), industry (panels **e-f**) and transport (panels **g-h**) by energy carrier in the *LeastCost-NZ* and *FosFree-NZ* scenarios.

Evolution and Composition of Electricity Generation and Demand

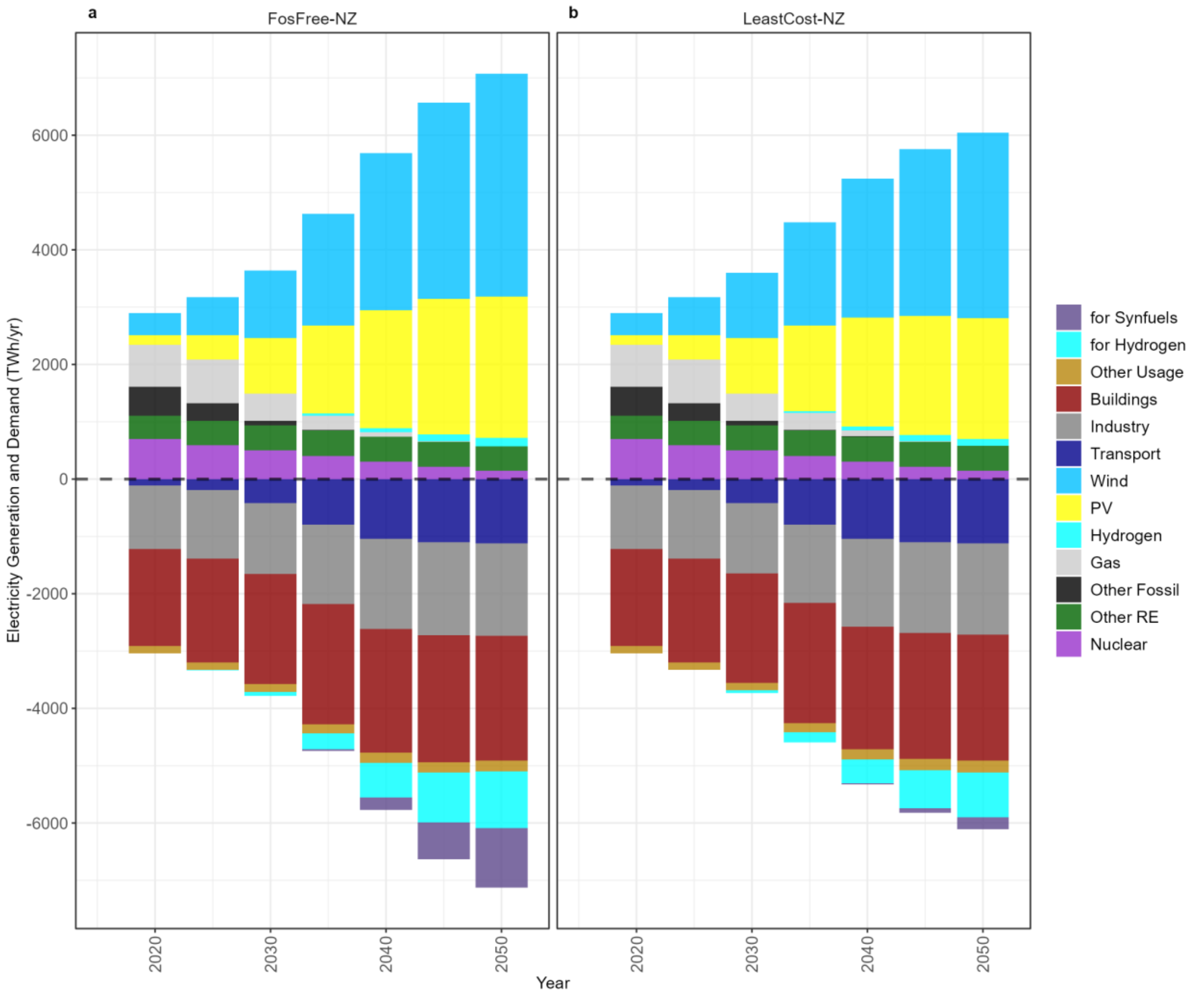

Supplementary Figure 2: EU electricity generation is shown across technologies as positive bars and electricity demand is shown across sectors as negative for the *FosFree-NZ* (panel **a**) and the *LeastCost-NZ* (panel **b**) scenarios. *Wind* refers to the total of offshore and onshore wind generation, *PV* refers to the generation from solar photovoltaics, *Other RE* refers to the generation from other renewable sources (hydropower, geothermal, biomass), *Gas* refers to generation from natural gas, *Other Fossil* refers to generation from coal or oil. *Other Usage* refers to demand by other energy or carbon management technologies including centralized heat pumps and carbon capture and storage facilities.

Evolution and Composition of Fossil Fuel Demand

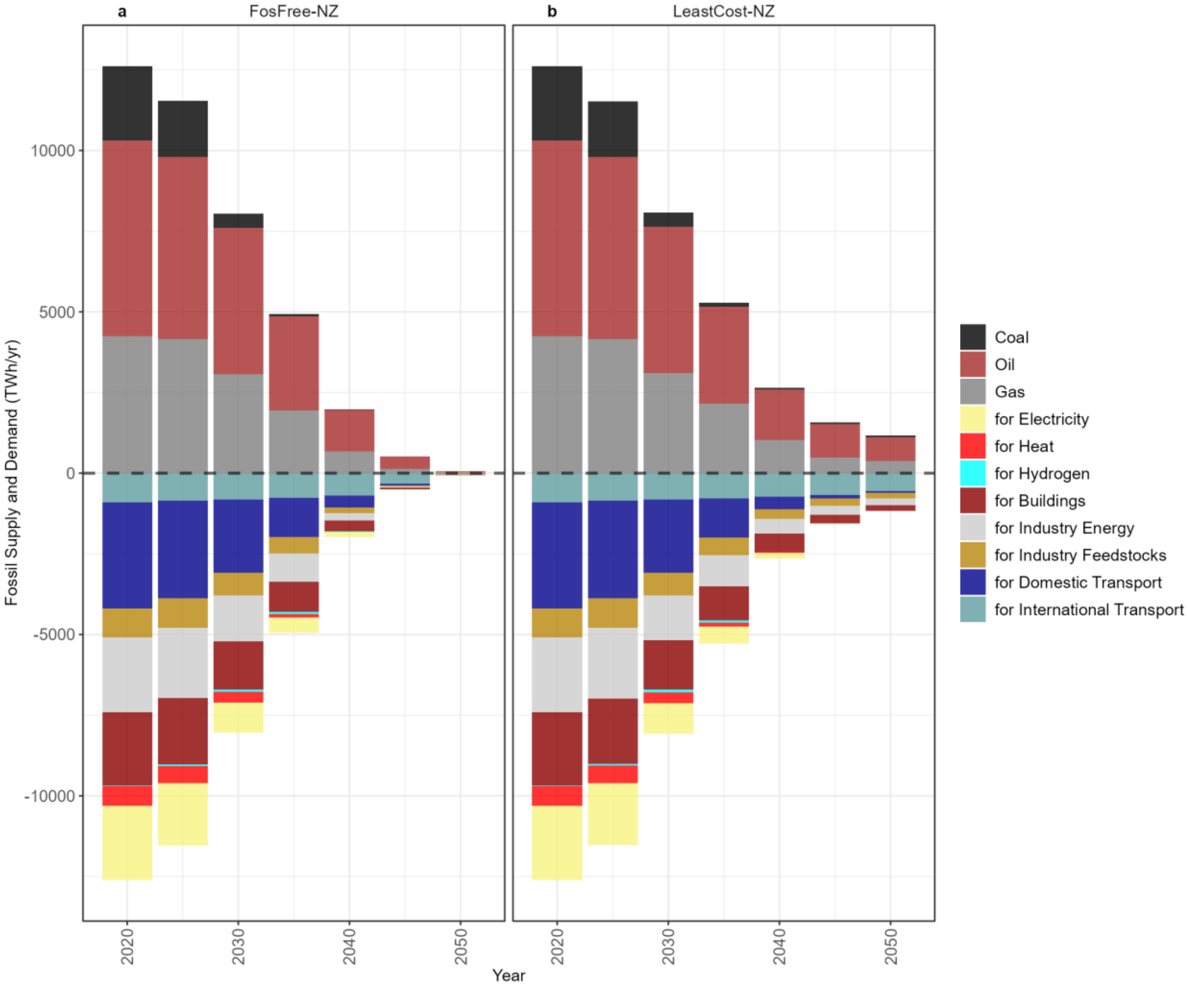

Supplementary Figure 3: EU fossil fuel supply is shown by energy carrier (coal, gas, oil) as positive bars and fossil fuel demand is shown across sectors or energy carriers fossil fuels are converted to in the FosFree-NZ (panel **a**) and the LeastCost-NZ (panel **b**) scenarios. Note that demand is shown in terms of primary energy fossil fuel input. *Heat* refers to the demand for producing heat for district heating networks. *Industry Energy* refers to the industrial demand for energy-related purposes (e.g. industrial heat and steam generation), while *Industry Feedstocks* refers to the industrial material demand of fossil hydrocarbons (e.g. to produce plastics).

Evolution and Composition of Hydrogen Supply and Demand

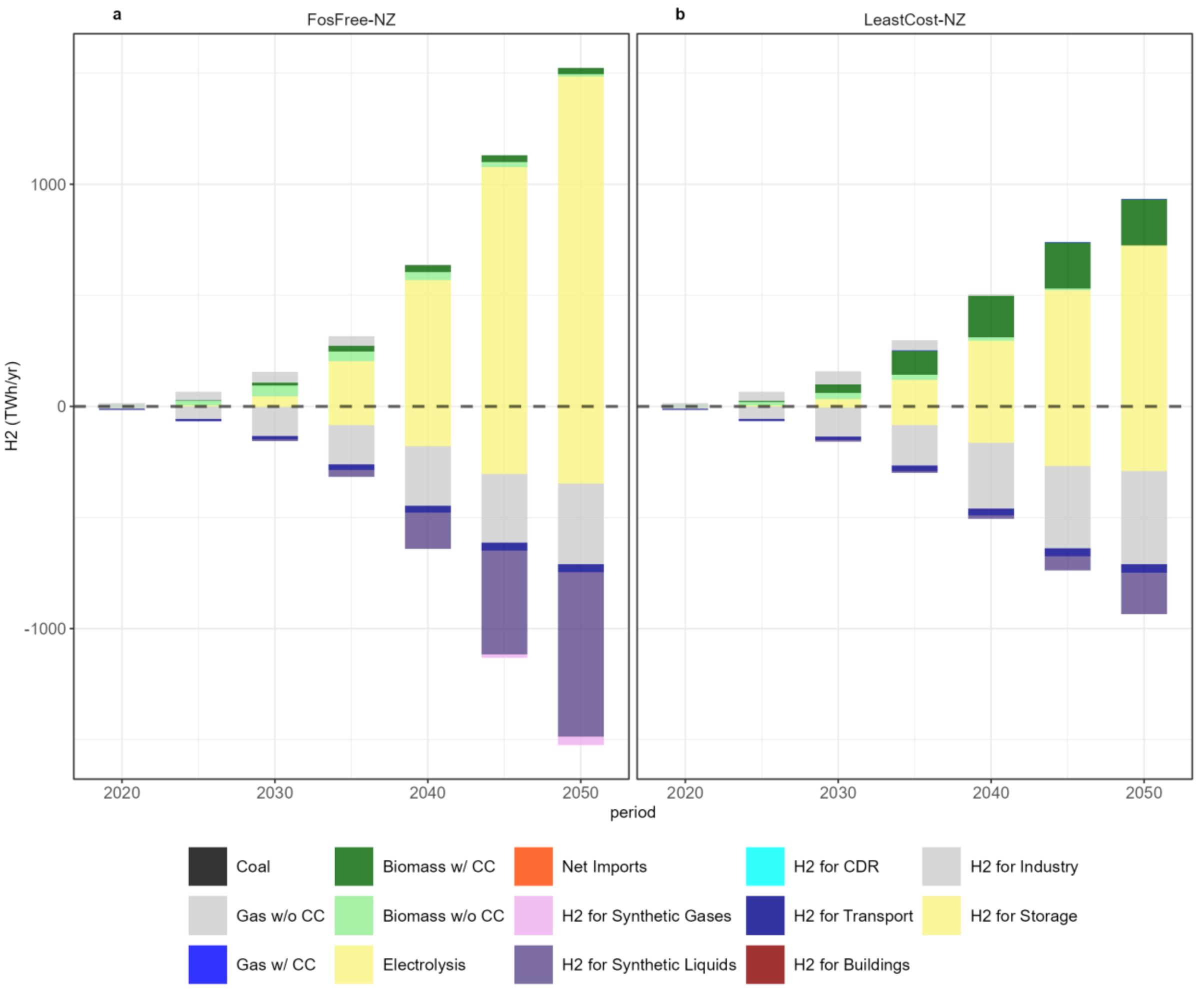

Supplementary Figure 4: EU Hydrogen supply and demand in the *FosFree-NZ* (panel **a**) and the *LeastCost-NZ* (panel **b**) scenarios. Supply is differentiated across production technologies as positive bars and demand is differentiated across sectors as negative bars.

Demand for Hydrocarbon Fuels in Final Energy by 2050

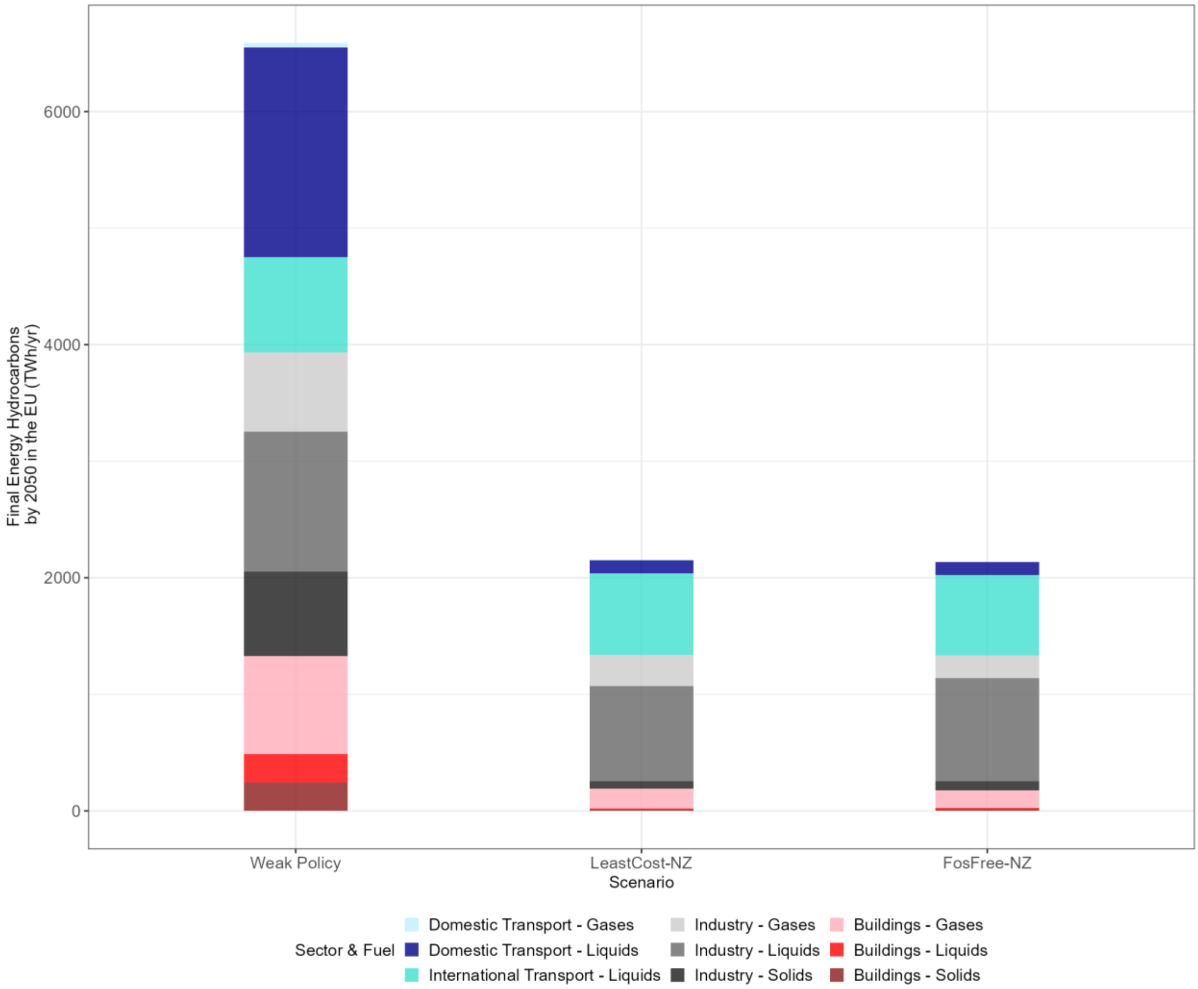

Supplementary Figure 5: EU final energy hydrocarbon demand is differentiated by type of fuel (solids, liquids, gases) and demand sector (domestic transport, international transport, industry, buildings). Next to the two net-zero scenarios *LeastCost-NZ* and *FosFree-NZ*, the *Weak Policy* scenario is shown, which represents a scenario with a continued low CO_2 price (see Methods section).

Evolution and Composition of Total Greenhouse Gas Emissions

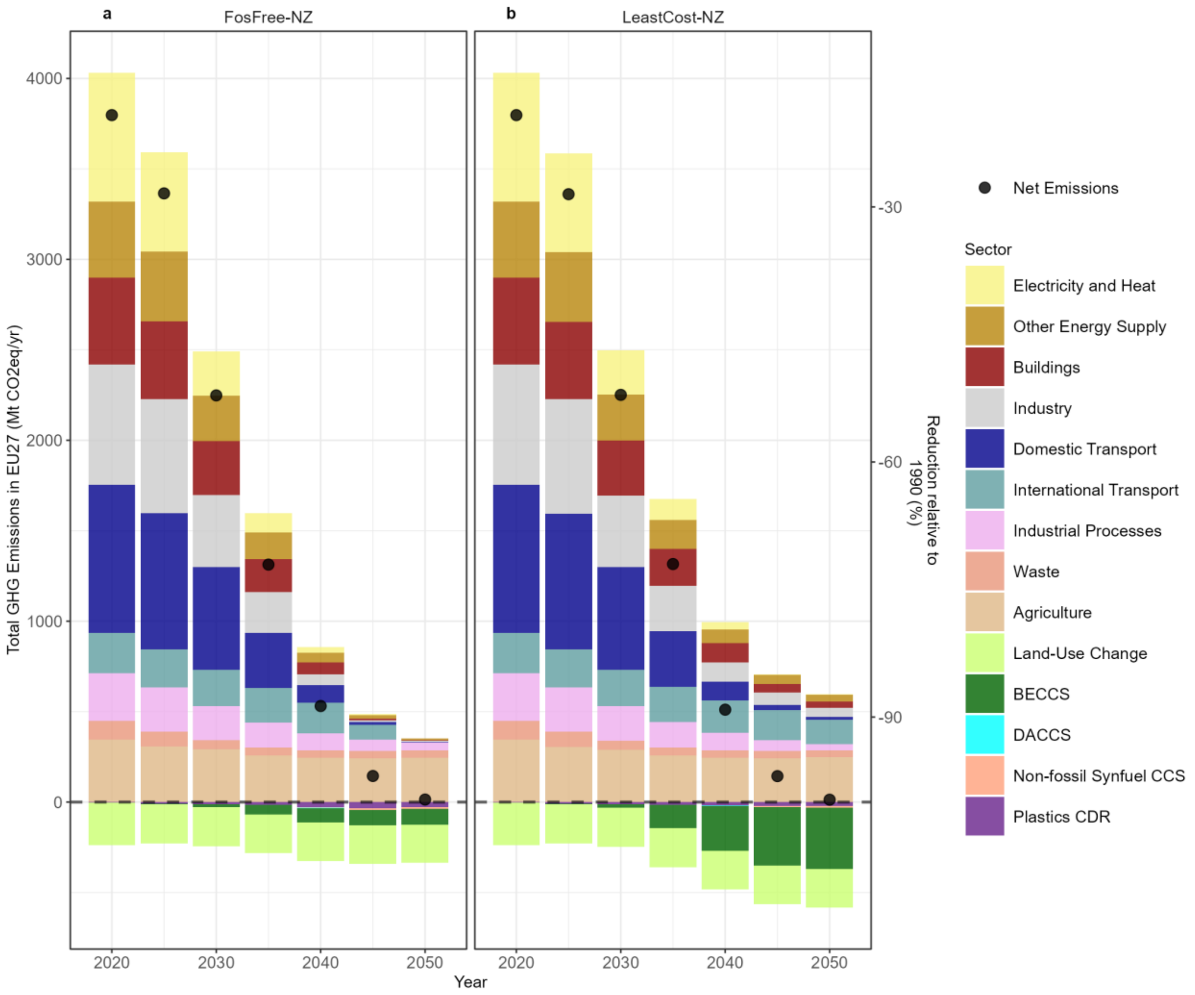

Supplementary Figure 6: Total greenhouse gas emissions in the EU up to 2050 shown across sectors in the *FosFree-NZ* (panel **a**) and *LeastCost-NZ* (panel **b**) scenarios. Negative bars refer to negative emissions from carbon dioxide removal options. The second y-axis shows the emissions relative to the reference year of the EU emissions targets in 1990. The black dots represent net emissions. *Other Energy Supply* refers to emissions from energy transformation technologies outside the electricity and heat sector such as oil refineries. *BECCS* refers to negative emissions from bioenergy with carbon capture and storage. *DACCS* refers to negative emissions from direct air capture with carbon capture and storage. *Non-fossil Synfuel CCS* refers to negative emissions via storing CO_2 from the combustion of carbon-neutral synthetic fuels. *Plastics CDR* refers to negative emissions from storing non-fossil CO_2 in permanently utilized or landfilled plastics.

Evolution of Total Carbon Dioxide Removal

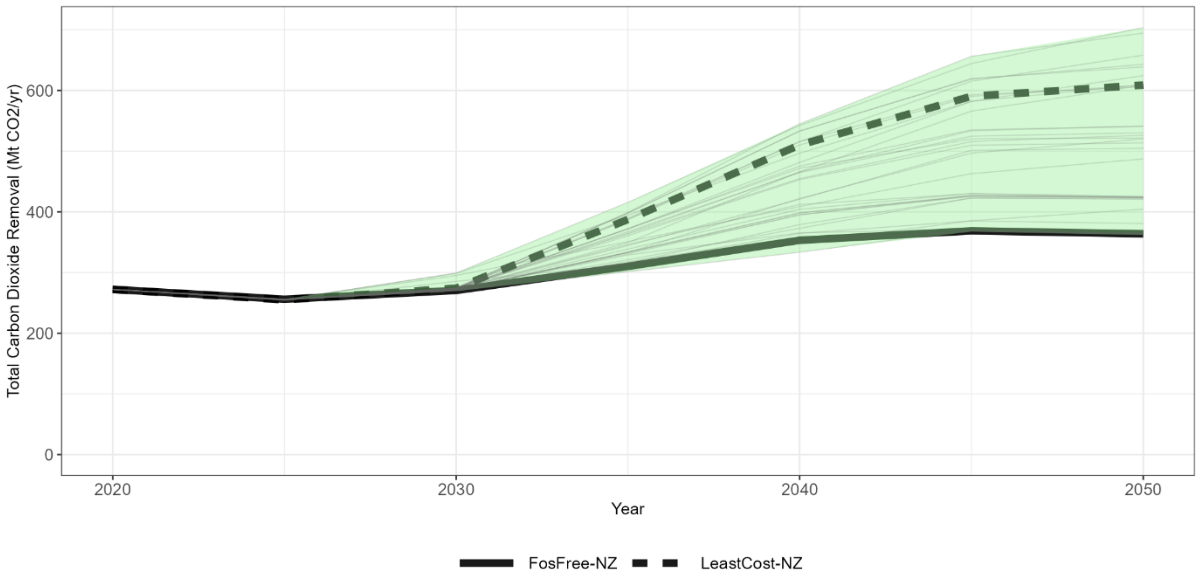

Supplementary Figure 7: Carbon dioxide removal in the EU across the *LeastCost-NZ* and the *FosFree-NZ* scenarios (thick lines) as well as all other net-zero scenarios of this study including sensitivity scenarios (thin lines and funnel, see Supplementary Table 1). This includes the land carbon sink, which generates negative emissions in historical time steps as well as all novel carbon dioxide removal options shown in Supplementary Figure 6.

Composition of Biomass Supply and Demand by 2050

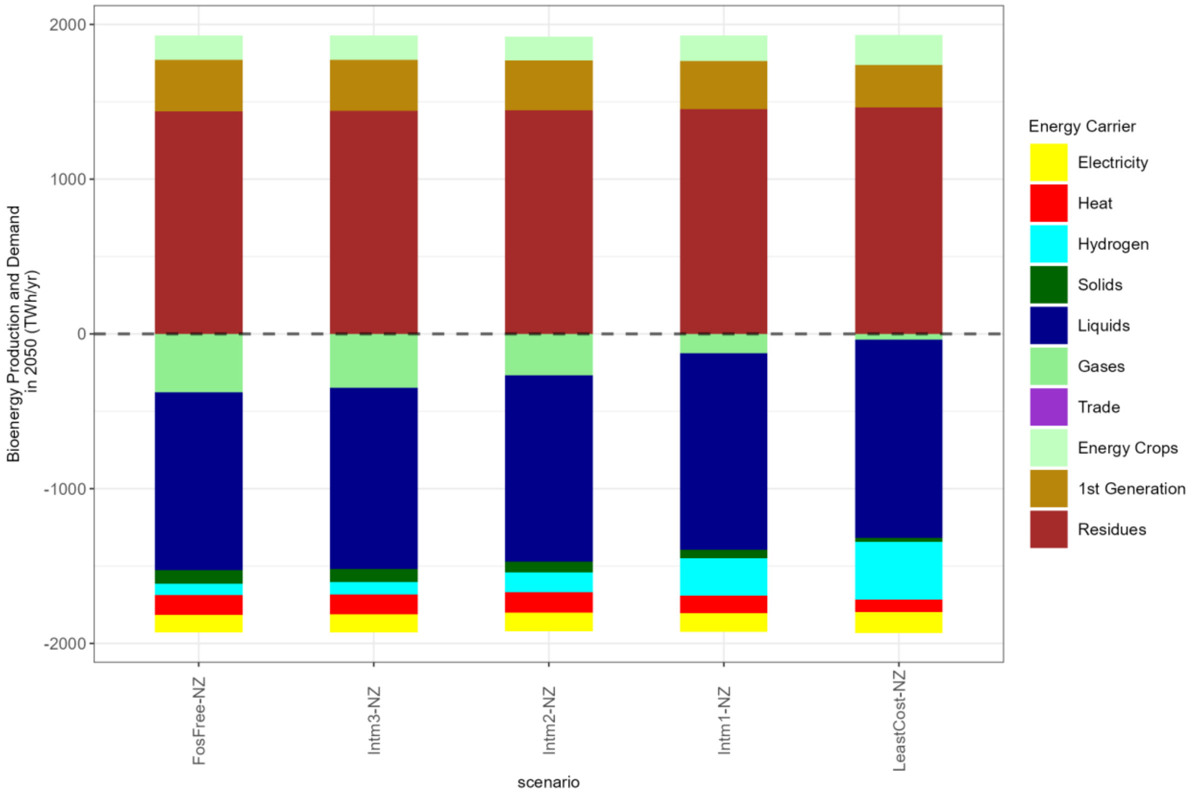

Supplementary Figure 8: EU biomass supply and demand in 2050 across the standard net-zero scenarios. Biomass supply is differentiated across feedstocks as positive bars (residues, 1st generation biomass and energy crops) and biomass demand is differentiated across sectors as negative bars. Note that biomass demand for solids, liquids and gases represents bioenergy used in the energy demand sectors (buildings, industry, transport), while biomass for electricity, heat (used for district heating) and hydrogen represents bioenergy used in the energy supply sector.

Carbon Capture, Storage and Utilization

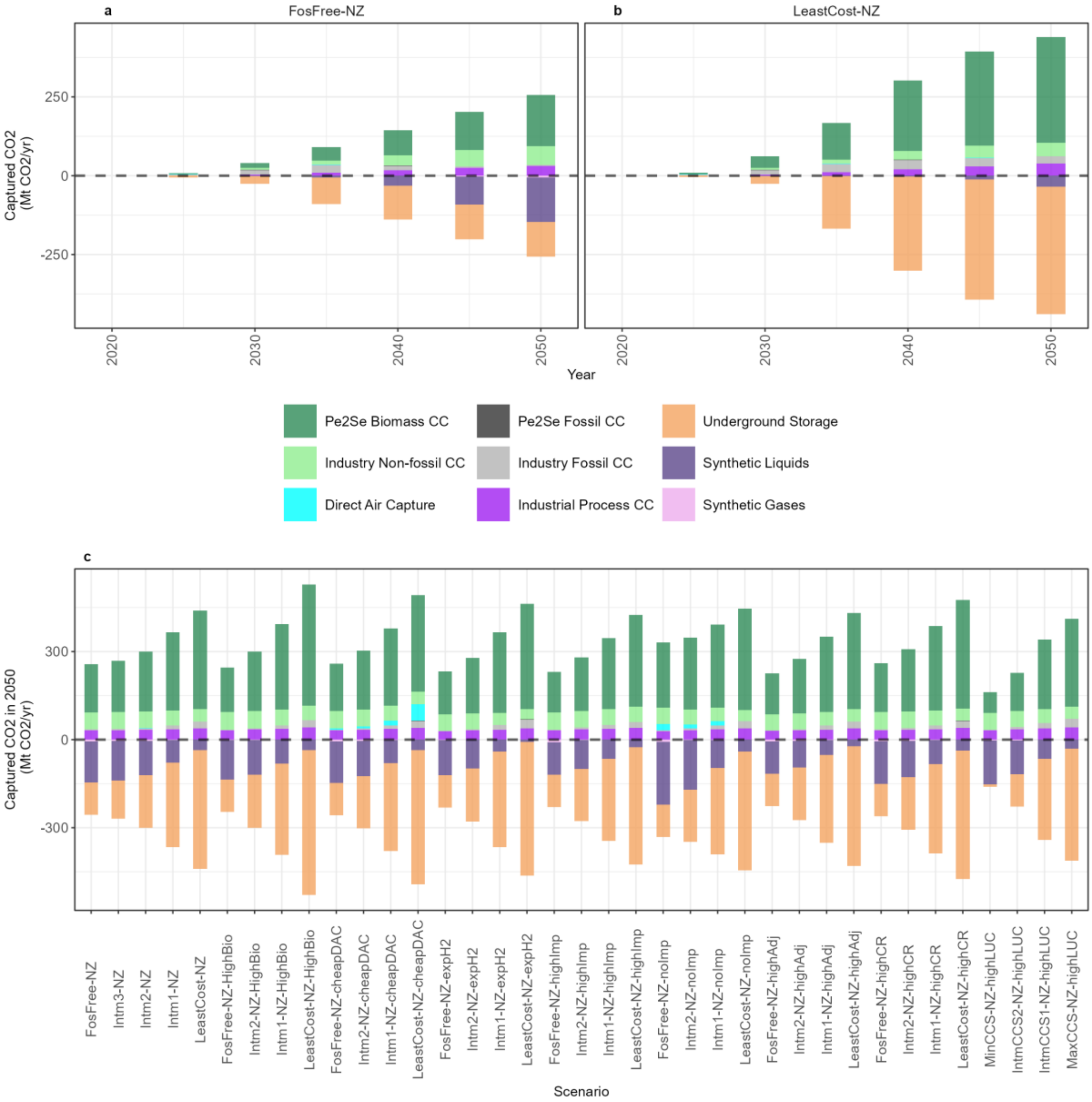

Supplementary Figure 9: a-b Evolution of captured carbon flows in the *FosFree-NZ* (panel **a**) and the *LeastCost-NZ* (panel **b**) scenarios up to 2050 in the EU by carbon source as positive bars and utilization as negative bars. Carbon capture (CC) is differentiated across the following sources: *Pe2Se Biomass CC* refers to biogenic carbon captured from energy conversion processes (primary to secondary energy conversions). In the scenarios, this is mostly carbon captured from the production of liquid biofuels. *Pe2Se Fossil CC* refers to fossil carbon captured from energy conversion processes. *Industry Non-fossil CC* and *Industry Fossil CC* refer to non-fossil and fossil carbon captured in industry for energy-related purposes respectively. *Industrial Process CC* refers to fossil carbon captured from industrial processes (carbon that would otherwise be accounted as industrial processes emissions). The utilization of carbon is differentiated across the following purposes: *Underground storage* refers to injection of CO_2 into geological storage reservoirs and *Synthetic Liquids* and *Synthetic Gases* refer to carbon used for the production of liquid (e-fuel) or gaseous fuels (e-gas) respectively. **c** As in **a** and **b** but showing only the 2050 state across all net-zero scenarios of this study including sensitivity scenarios. Note that in *CheapDAC* scenarios some deployment of direct air capture (cyan) is forced. In the *FosFree-NZ-cheapDAC* scenario, for instance, direct air capture is only deployed up to the forced amount such that this deployment is not an outcome of the optimization. However, the higher deployment levels observed in the *LeastCost-NZ-cheapDAC* scenario are an endogenous optimization outcome.

Marginal Abatement Costs and Fuel-switching CO_2 Prices Across Sensitivity Scenarios

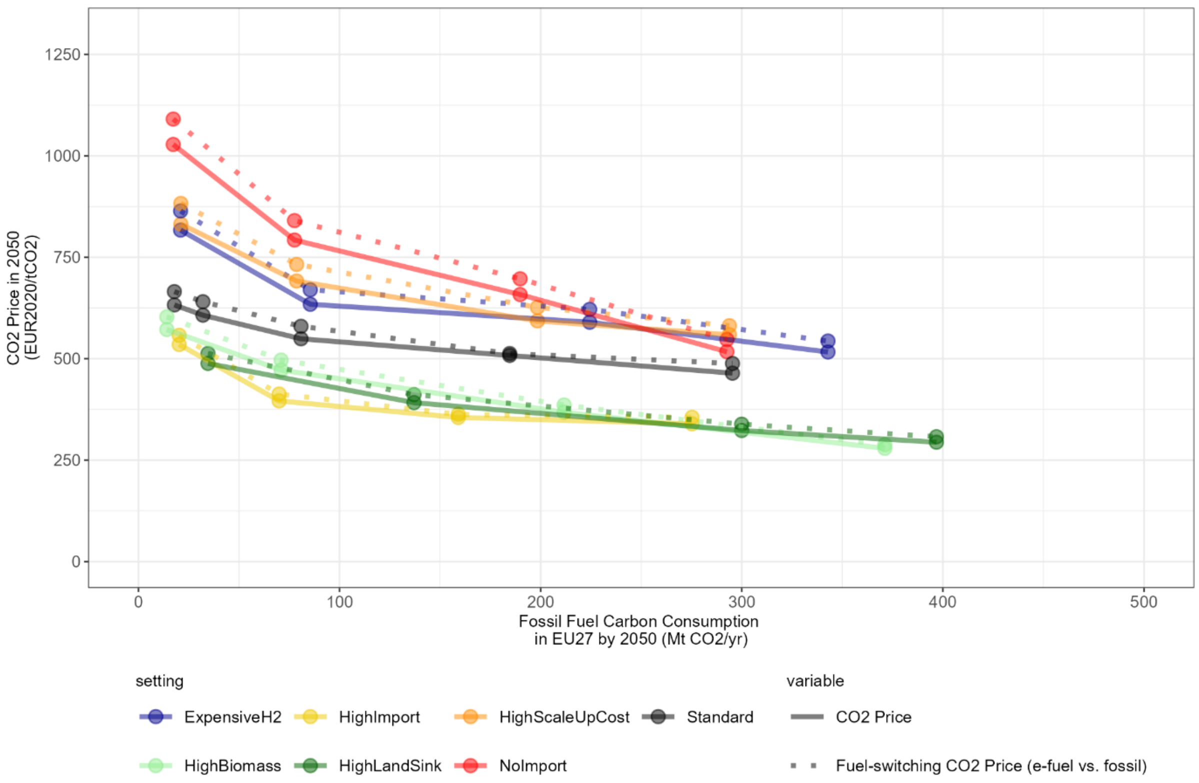

Supplementary Figure 10: Marginal abatement cost by 2050 in the EU on the y-axis against fossil fuel carbon consumption on the x-axis across standard scenarios and selected sensitivity scenarios (solid lines) as in Figure 5a. Moreover, corresponding fuel-switching CO_2 prices for replacing fossil liquid fuels by e-fuels derived from shadow prices of the model are shown (dotted lines). For the definition of sensitivity scenarios see Supplementary Table 2. For the calculation of fuel-switching CO_2 prices see Methods section.

Secondary Energy Prices of Fossil Liquid Fuels and E-fuels

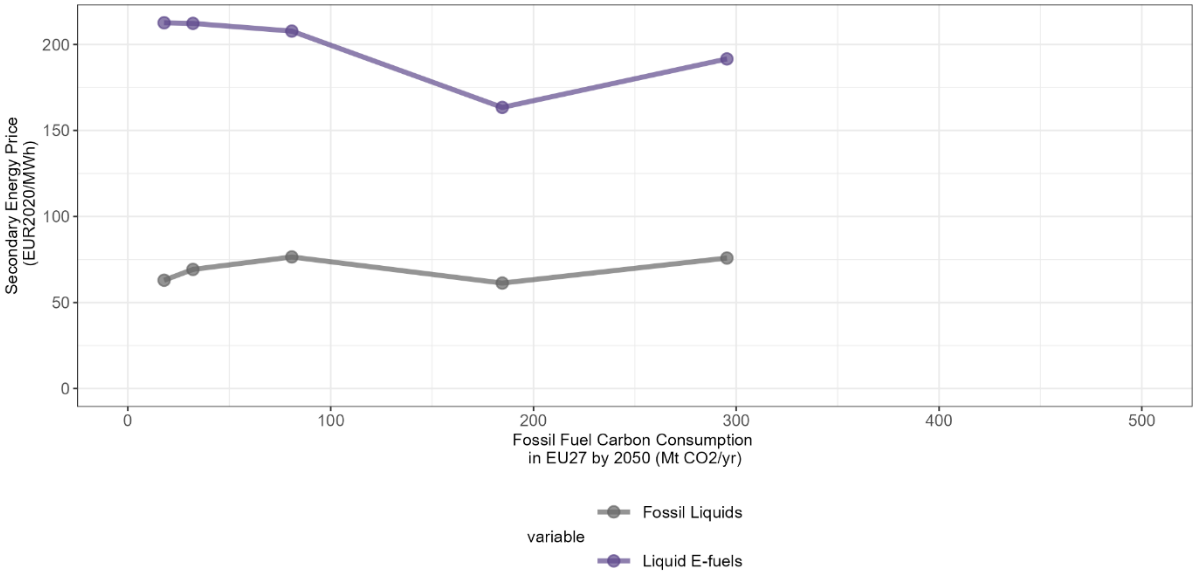

Supplementary Figure 11: Secondary energy prices in 2050 on the y-axes against fossil fuel carbon consumption on the x-axis across standard net-zero scenarios. The prices represent the 2050 EU-wide averaged secondary energy price (producer level, i.e. before transport and taxes) of fossil liquid fuels (grey line) and liquid e-fuels (purple). Only regions with more than 1 TWh yr^{-1} of fossil liquids demand were included in the averaging as model prices are not reliable for very low quantities.

Cost Assessments Across Further Sensitivity Scenarios

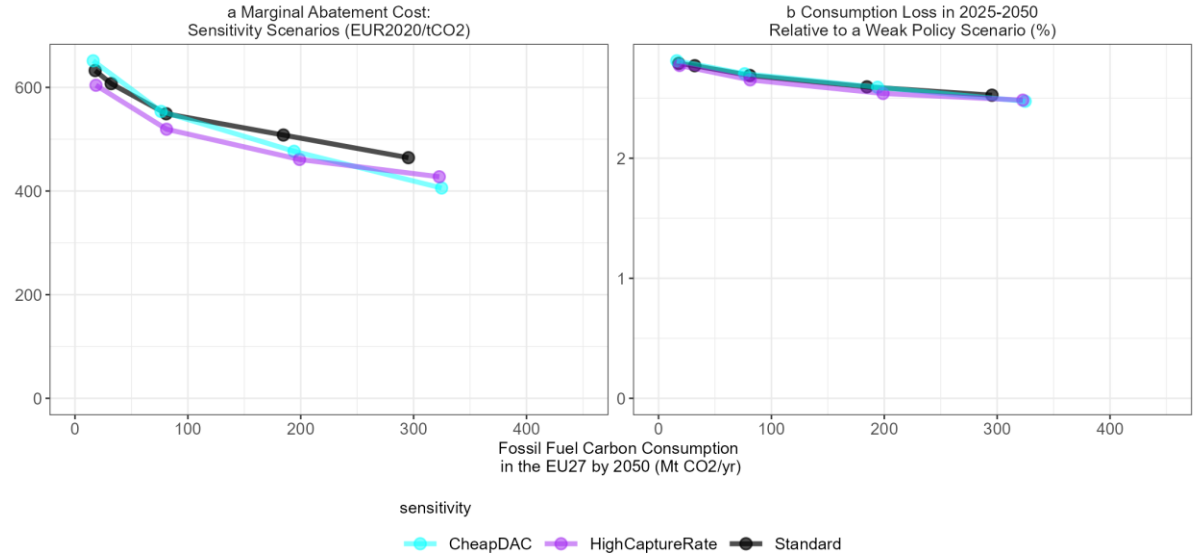

Supplementary Figure 12: a EU marginal abatement costs across standard scenarios (black line) and sensitivity scenarios with high CO_2 capture rate (*HighCaptureRate*, in purple) and low cost of direct air capture (*CheapDAC*, in cyan). **b** EU aggregate 2025-2050 consumption losses (discounted at 3% per year) relative to the *Weak Policy* scenario (see Methods section) for the same scenarios. The respective x-axes show fossil fuel carbon consumption in 2050, i.e. the total fossil carbon input into the energy system at net-zero.

Final Energy Prices of Relevant Energy Carriers

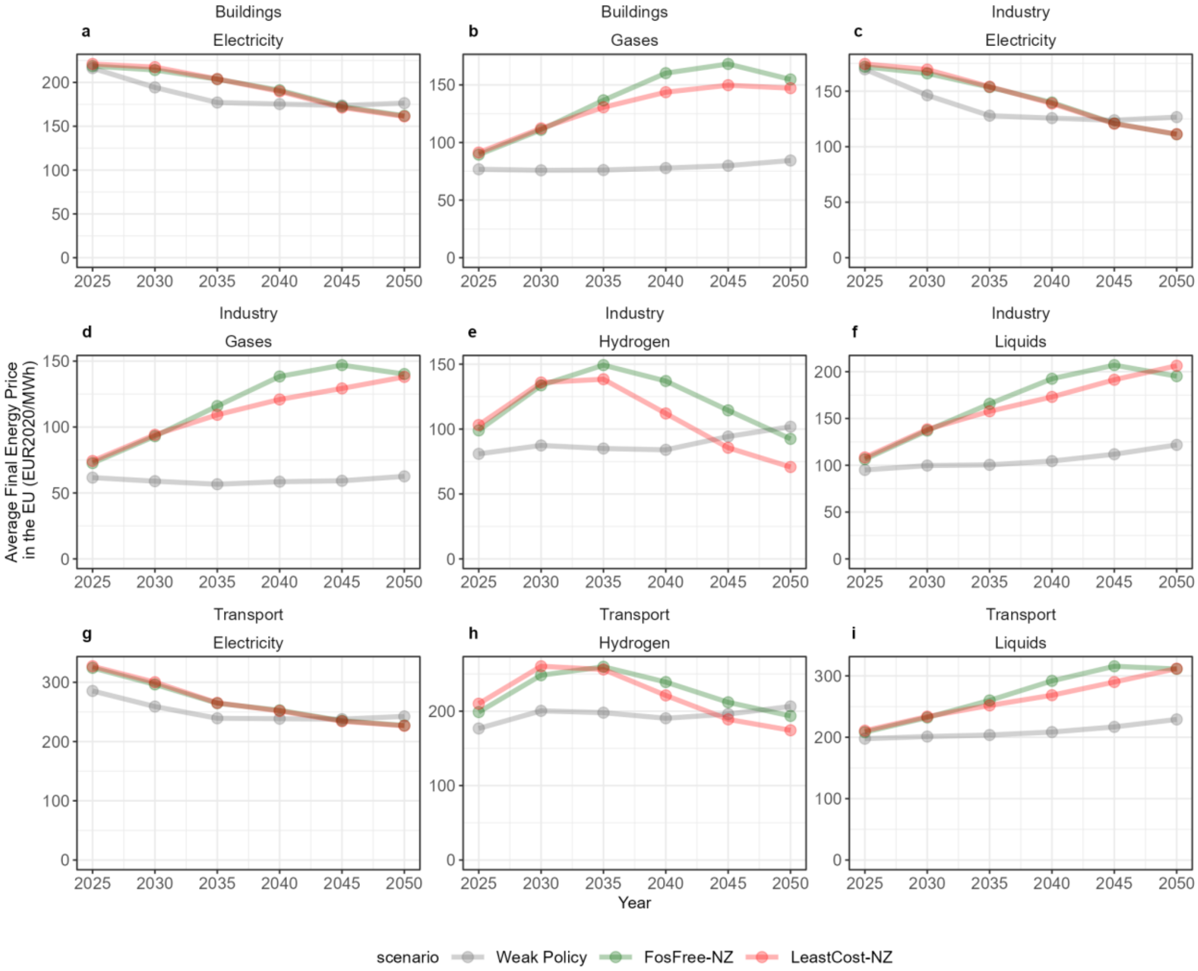

Supplementary Figure 13: Evolution of EU-wide averaged final energy prices (i.e. including transmission and distribution as well as taxes) for electricity, gases, liquid fuels and hydrogen in the buildings (panels **a-b**), industry (panels **c-f**) and transport (panels **g-i**) for the *Weak Policy*, *FosFree-NZ* and *LeastCost-NZ* scenario. These prices correspond to prices faced by energy consumers in the respective sector. The *Weak Policy* scenario represents a scenario with low ambition of EU climate policy as of before the regulation of the European Green Deal that fails to reach European climate targets for 2030 and 2050 (see Methods section).

Supplementary Tables

Overview about EU Net-zero Scenarios Investigated by this Study

Scenario Setting	Maximum CCS: 10 $MtCO_2$ yr^{-1}	Maximum CCS: 110 $MtCO_2$ yr^{-1}	Maximum CCS: 130 $MtCO_2$ yr^{-1}	Maximum CCS: 180 $MtCO_2$ yr^{-1}	Maximum CCS: 350 $MtCO_2$ yr^{-1}	Maximum CCS: 2000 $MtCO_2$ yr^{-1}
**Standard**		FosFree-NZ (core scenario)	Intm3-NZ	Intm2-NZ	Intm1-NZ	LeastCost-NZ (core scenario)
**HighBiomass**		FosFree-NZ-HighBio		Intm2-NZ-HighBio	Intm1-NZ-HighBio	LeastCost-NZ-HighBio
**CheapDAC**		FosFree-NZ-CheapDAC		Intm2-NZ-CheapDAC	Intm1-NZ-CheapDAC	LeastCost-NZ-CheapDAC
**ExpensiveH2**		FosFree-NZ-expH2		Intm2-NZ- expH2	Intm1-NZ- expH2	LeastCost-NZ-expH2
**HighImport**		FosFree-NZ-HighImp		Intm2-NZ-HighImp	Intm1-NZ-HighImp	LeastCost-NZ-HighImp
**NoImport**		FosFree-NZ-noImp		Intm2-NZ-noImp	Intm1-NZ-noImp	LeastCost-NZ-noImp
**HighScaleUpCost**		FosFree-NZ-HighScaleUpCost		Intm2-NZ-HighScaleUpCost	Intm1-NZ-HighScaleUpCost	LeastCost-NZ-HighScaleUpCost
**HighCaptureRate**		FosFree-NZ-HighCaptureRate		Intm2-NZ-HighCaptureRate	Intm1-NZ-HighCaptureRate	LeastCost-NZ-HighCaptureRate
**HighLandSink**	FosFree-NZ-HighLandSink	Intm2-NZ-HighLandSink			Intm1-NZ-HighLandSink	LeastCost-NZ-HighLandSink

Supplementary Table 1: Overview about EU net-zero scenarios investigated by this study. There are two main dimensions of scenario assumptions: First, variations of assumptions related to maximum possible carbon capture and storage (CCS) injection in the EU to generate net-zero scenarios with different amounts of residual fossil energy use (columns). Second, variations of other technology-related assumptions that generate the sensitivity scenarios of the *HighBiomass*, *CheapDAC*, *expensiveH2*, *HighImport, NoImport*, *HighScaleUpCost, HighCaptureRate* and *HighLandSink* categories (rows). The *HighLandSink* sensitivity scenarios have lower CCS injection limits in the *FosFree-NZ and Intm2-NZ* case since the scenarios should be similar in terms of residual fossil fuel demand relative to their counterparts in the other categories. A higher land sink allows for lower CCS deployment at net-zero at the same level of residual fossil fuel demand. For details on the assumptions in the sensitivity scenarios, please refer to Supplementary **Error! Reference source not found.**.

Scenario Assumptions in Standard and Sensitivity Scenarios

Sensitivity scenarios	Parameter	Assumption in standard scenarios	Assumption in respective sensitivity scenarios	Description
**HighBiomass**	EU bioenergy potential	7.5 EJ yr^{-1} primary energy biomass	12.5 EJ yr^{-1} primary energy biomass	Based on low scenario (standard) and reference scenarios in Ruiz et al.[1].
**CheapDAC**	DAC learning rate (investment cost reduction for doubling of cumulative capacity)	15%	25%	Sensitivity with optimistic DAC cost assumptions. The standard learning rate of 15% also used for renewable technologies is increased to 25%.
	DAC energy demand:	5.8 GJ tCO_2^{-1} heat, 1.4 GJ tCO_2^{-1} electricity	2.9 GJ tCO_2^{-1} heat, 0.7 GJ tCO_2^{-1} electricity (-50% rel. to default)	Default DAC energy demand based on Beuttler et al.[2] is halved.
	DAC deployment	No minimum DAC capacity	At least 100 $MtCO_2$ yr^{-1} global capacity in 2040	Force some global DAC deployment to have lower CAPEX in the EU in 2050.
**ExpensiveH2**	Electrolysis floor costs	100 € $kW(el)^{-1}$	300 € $kW(el)^{-1}$	Increase floor cost of electrolysis learning parameterization.
	Electrolysis electricity price	20-35 € $MWh(el)^{-1}$	30-50 € $MWh(el)^{-1}$	To capture uncertainty about electricity prices. Note: The price is endogenous. Ranges are indicative. The parameter we vary is the slope of the price duration curve.
**HighImport**	E-fuel imports	50% of e-liquids demand is always imported.	E-liquids and e-gas can be imported at fix price of 150 € MWh^{-1} in 2050 to the EU.	Sensitivity with abundant imports at fixed price at a level indicated by Hampp et al.[3] and Pfennig et al.[4]
**NoImport**	E-fuel imports	50% of e-liquids demand is always imported.	No e-liquids or e-gas imports.	Sensitivity without e-fuel imports.
**HighScaleUpCost**	Investment cost mark-up depending on technology growth rate	Cost mark-up depending on square of difference in capacity additions between time steps	Double cost-mark-up for all technologies relative to default parameterization.	Sensitivity to capture uncertainty around e-fuel up-scaling speed and costs.
**HighCaptureRate**	CO_2 capture rate for gasification technologies	90% of carbon from biomass gasification can be captured.	99% of carbon from biomass gasification can be captured.	Sensitivity to represent uncertainty about CO_2 capture technologies.
**HighLandSink**	Land sink in 2050	240 $MtCO_2$ yr^{-1} negative emissions via land sink.	370 $MtCO_2$ yr^{-1} negative emissions via land sink.	Sensitivity with higher land sink assumptions motivated by EU 2030 land sink target (310 $MtCO_2$ yr^{-1}) and the long-term range by Pilli et al.[5] (100 to 400 $MtCO_2$ yr^{-1}).

Supplementary Table 2: Scenario Assumptions in Standard and Sensitivity Scenarios. The table lists the category of sensitivity scenario (first column), the parameter changed in the model (second column), the default assumption in a standard scenario (third column), the assumption made in the respective sensitivity scenario (fourth) column and a description of this change (fifth column).

Supplementary References

1. Ruiz, P. *et al.* ENSPRESO - an open, EU-28 wide, transparent and coherent database of wind, solar and biomass energy potentials. *Energy Strategy Reviews* **26**, 100379 (2019).
2. Beuttler, C., Charles, L. & Wurzbacher, J. The Role of Direct Air Capture in Mitigation of Anthropogenic Greenhouse Gas Emissions. *Front. Clim.* **1**, 10 (2019).
3. Hampp, J., Düren, M. & Brown, T. Import options for chemical energy carriers from renewable sources to Germany. *PLoS ONE* **18**, e0262340 (2023).
4. Pfennig, M. *et al.* Global GIS-based potential analysis and cost assessment of Power-to-X fuels in 2050. *Applied Energy* **347**, 121289 (2023).
5. Pilli, R., Alkama, R., Cescatti, A., Kurz, W. A. & Grassi, G. The European forest Carbon budget under future climate conditions and current management practices. Preprint at https://doi.org/10.5194/bg-2022-35 (2022).
